# QSAR Models for Reproductive Toxicity and Endocrine Disruption Activity

**DOI:** 10.3390/molecules15031987

**Published:** 2010-03-22

**Authors:** Marjana Novič, Marjan Vračko

**Affiliations:** National Institute of Chemistry, Hajdrihova 19, 1000 Ljubljana, Slovenia; E-Mail: marjana.novic@ki.si (M.N.)

**Keywords:** reproductive toxicity, modeling, CAESAR program, counter propagation neural networks

## Abstract

Reproductive toxicity is an important regulatory endpoint, which is required in registration procedures of chemicals used for different purposes (for example pesticides). The *in vivo* tests are expensive, time consuming and require large numbers of animals, which must be sacrificed. Therefore an effort is ongoing to develop alternative *In vitro* and *in silico* methods to evaluate reproductive toxicity. In this review we describe some modeling approaches. In the first example we describe the CAESAR model for prediction of reproductive toxicity; the second example shows a classification model for endocrine disruption potential based on counter propagation artificial neural networks; the third example shows a modeling of relative binding affinity to rat estrogen receptor, and the fourth one shows a receptor dependent modeling experiment.

## 1. Introduction

The term reproductive toxicity indicates anything disturbing the reproductive process of organisms. It includes adverse effects on the reproductive ability of individuals such as alternation of sexual organs and behavior, and the developmental toxicity of offspring. Different experimental methods, which are available for assessing of the reproductive toxicity, are standardized and described in guidelines issued from different agencies. The OECD library reports several tests related to reproductive toxicity. The documents OECD TG 422 and TG 421 describe the screening test, which can be used to get initial information on reproductive or developmental toxicity and can be used at an early stage of assessing of chemicals [1,2]. It does not provide the complete information on developmental toxicity of tested chemicals, what means that negative results do not necessarily indicate the safety of a chemical. On the other hand, any positive results are useful for initial hazard assessment and the priority setting for further testing. More informative are one-generation and two-generation tests, which are described in documents TG 415 and TG 416, respectively [3,4]. The tests provide the general information about the functioning of reproductive systems of males and females including information on gonadal function, the oestrus cycle, mating behaviour, conception, gestation, parturition, lactation, and weaning. The tests also provide the information about effects on growth and development of offspring. For specific functional deficiency furher tests are available like Developmental Neurotoxicity Study [5], Uterotropfic Bioassays for (anti) Estrogenic Effects [6], and Hersberger Bioassay for (anti) Androgenic Effects [7].

The tests are among the most expensive ones and require sacrifice of a large number of animals, therefore there considerable interest and efforts have been invested into developing alternative *In vitro* and *in silico* methods [8,9,10,11,12,13,14,15,16]. Quantification of endpoints represents a problem in evaluation of reproductive toxicology. The OECD documents TG 421, TG 422, and TG 414 describe the experimental conditions for animal tratment and evaluation of reproductive toxicity effects. The clinical observations are often given in descriptive form, for example, when the target organs of sacrificed animals are examined on histopatology, or, the data are given in tabular form as numbers of dead offspring, body weight, mating behaviour, the animals showing intoxication, etc. For *in silico* modeling purposes all the data must be expressed in numerical form, which enables the treatment with standard statistical tools. On the other hand, for the regulatory purposes the results are used in classification schems. The United Nations (UN) Globaly Harmonized System of Classification and Labeling of Chemicals (GHS UN2003) provides a classification scheme where chemicals are classified into one of two categories (1, 1A, 1B, and 2). Category 1 defines substances, which are known or presumed human reproductive toxicants. In the category 1A are substances that are known to have produced an adverse effect on reproductive ability or capacity or on development in human. The classification is based on evidences on humans. In the category 1B are subatances that are presumed to produce an adverse effect on reproductive ability or capacity or on development in human. The classification is based on evidences from animal experiments. To category 2 belong substances that are suspected to be human reproductive or developmental toxicants. For these compounds there is some evidence from human or experimental animals, however, the evidence is not sufficiently convincing to place the substance in category 1. The EU Member Countries use the classification system described in Annex VI to Commision Directive 2001/59/EC (Anon 2001). The reproductive toxicity assigned to chemicals harmful to fertility and developmental processes is divided into three categories with respect to the level of risk. Category 1 includes substances that are known to impair fertility in humans and that are known to cause developmental toxicity in humans. They are placed into this category if there is sufficient evidence of their toxicity. Category 2 comprises substances which should be regarded as if they impair fertility in humans or cause developmental harm in humans. They are assigned to this category if there is sufficient evidence on their toxicity, generally on the basis of animal experiments. Category 3 includes substances that cause concern for human fertility or may have developmental toxic effects. They are placed into this category generally on the basis of results of animal studies, however, the evidence is insufficient to place them in categories 1 or 2. The FDA has a classification system with five categories; category A means negative human studies, category B means negative animal studies and no human studies executed or positive animal studies and negative human studies, category C means positive animal studies and no human studies or no studies at all. Category D means positive human studies, and category X means animal or human studies show abnormalities and/or evidence of foetal risk based on human experience.

It is clear that the data of such a structure are poorly suitable for QSAR modeling. In the sections that follow we present some strategies for approaching this problem. In Section 3 we present four examples in more details. First, we present the CAESAR model for prediction of reproductive toxicity. Second, we show the classification model for endocrine disruptors, third, we show an example of modeling of estrogen binding affinity, and fourth, we show an example where the information on estrogen receptors is a part of modeling.

## 2. Modeling Strategies

There are several ways to model reproductive toxicity using (Q)SAR methodology. In the SAR approach one is focused on chemical structures having in mind chemical categories and similarity among molecules. An OECD definition of a chemical category reads: “A chemical category is a group of chemicals whose physicochemical and toxicological properties are likely to be similar or follow a regular pattern as a result of structural similarity….” [8]. A categorisation can be done by experts (read across) or by using of computer classification algorithms. Another way is the standard QSAR approach where the models are built on a clearly defined activity related to a specific target, which are related to developmental toxicity. The basic properties are related to transport and distribution of substances like bariers for transport from blood to testis, placenta, brain, breast milk, etc. Often studied properties are binding affinities to sex hormone receptors (estrogen receptors, androgen receptors) and for thyroid hormone receptors. A review over current status of structural based methods for estimation of reproductive toxicity is reported in [9]. The report includes among others the information on training sets for DEREK, TOPKAT, MC4PC, PASS, HazardExpert, OSIRIS property explorer, and OECD (Q)SAR Application Toolbox. Most of the QSAR models, which are focused on specific target, have been developed for estrogen receptors. In [10] authors applied three methods, decision tree, and learning vector quantization and, for classification of 311 compounds as active or inactive for estrogen receptor. Structures were described with DRAGON descriptors. Authors report the best results for the k-nearest neighbour method. In [11] the authors propose a two-descriptor model for classification of compounds as estrogenically active or non-active compounds. The model is commented in terms of OECD principles for validation of QSAR models used for regulatory purposes [12]. In [13] the authors applied different chemometrical tools to analyse the endocrine activity of over 11,000 compounds. The model described in [14] was made on the basis of FDA/TERIS data base, which consists of 292 structurally and chemically diverse drug-like compounds. The authors applied logistic regression and CART modelling techniques for classification of compounds into two clases (toxicants, non-toxicants). Average sensitivity, specificity and accuracy for several tests are at about 60% for all three statistical parameters. The most important physico-chemical descriptors involved in the logistic models were: density, log P and molecular weight and in CART models: log P, HOMO energy, LUMO energy, hydrogen acceptor, hydrogen donor, and molar refractivity. In [15] a set of 27 conazoles was investigated with hierarchical clustering, principal component method and Kohonen networks. On the basis of cluster pattern analysis a classification was proposed for some conazoles. In [16,17] authors report *In vitro* and *in silico* approaches to the developmental and reproductive toxicity including the endocrine disruption. In [18] a data set of brominated compounds was examined with QSAR methods for their *In vitro* potency. In [19] 200 structurally diverse chemicals were categorized with decision trees and support vector machine techniques with respect to their androgen receptor binding affinity. Report [20] focuses on screening of 57,014 EINECS chemicals investigating teratogenic endpoints with data obtained from animal experiments, clinical data and epidemiological studies. A competitive binding to thyroid hormone transport protein transthyretin was studied on a set of fluorinated compounds in reference [21]. In [22] the common reactivity pattern approach (COREPA) was applied to study the relative binding affinity to estrogen receptor. In [23] 3D-QSAR models for three receptors, which mediate the endocrine disruption, are presented. In [24] the authors used principal component analysis to compress three estrogen activities into a single variable called Estrogen Activity Index, which was further used for QSAR modelling. The three activities resulted from receptor binding affinity assay, receptor gene assay and the cell proliferation assay. In [25] the authors report a model for estrogen receptor binding affinity developed in terms of OECD principles. A comparison of three different non-linear classification methods (least-square support vector machine, counter-propagation artificial neural network, and k nearest neighbour) for classification of estrogen-like chemicals is carried out in [26] using external validation set of 87 chemicals (prediction set) not included in the training set (232 chemicals). In [27] a review of some recent advances in the use of machine learning techniques in modeling estrogen-like chemicals is given. The authors discuss on the advantages and disadvantages of the machine learning algorithms and on the importance of the validation and performance assessment of the models and their applicability domains. In [28] two-step model for prediction of estrogen receptor binding affinity is presented. In [29] a virtual test kits for predicting harmful effects triggered by chemicals is presented. An overview of *in silico* methods in modelling of endocrine disruption is given in [30]. Different authors discussed the mechanisms of endocrine disruptions, the pharmacokinetic of compounds related to endocrine disruption, and discuss different QSAR models for assessing the endocrine disruption. A survey of (Q)SAR models, which are important in chemical regulation, is given in [31]. Its authors discussed the advantages and disadvantages of models, which are built on data sets of congeneric compounds, or on broader sets of non-congeneric compounds.

## 3. Examples

### 3.1. Prediction of Developmental Toxicity with CAESAR Model

Recently adopted European Chemical Regulation (REACH) envisages the wide use of computer assisted models for evaluation of chemical properties to replace *in vivo* and *In vitro* testing. Within the EU supported project CAESAR (Computer Assisted Evaluation of Substances According to Evaluation) the models for five regulatory ednpoints were developed and made publically available via the Internet [32] together with comments to five OECD principles for validation of (Q)SAR models used for regulatory purposes. The five regulatory endpoints are: bioconcentration factor, skin sensitization, mutagenicity, carcinogenicity, and developmental toxicity. The model for developmental toxicity was built on the data set of 292 compounds [14] described with 13 descriptors. In the CAESAR model a compound is classified as non-toxic if a compound is classified under FDA scheme to categories A or B and as toxic if it is classified to the categories C, D, or X. The classification result expressed as a binary value can not be directly used for categorisation of a chemical; rather it is an evidence, which can be used in the early stage of hazard assessment. Beside this result the model provides the structure of six compounds, which are the most similar to investigated compound. The training set consists of only 293 compounds and it is unlikely that an arbitrary compound hits close to a structure from the training set. However, the training set consists of very diverse compounds and therefore the prediction may have general validity.

Table 1 shows an example of predicting the developmental toxicity for four PAH compounds. Anthracene and fluoranthene are classified as toxicant, fluorene and triphenylene as non-toxicant. Additional information provided by the model is that the descriptors of triphenylene are out of model’s descriptor range. For each prediction the program displays the six most similar compounds from the data base. Comparing the sets for anthracene and fluorene in the Table 1 one can see that four compounds are the same. In fluorene the non-toxic diphenylhydramine and toxic alprazolam are replaced with toxic imipramine and amitriptyline. Predictions for fluoranthene and triphenylene are toxic and non-toxic, respectively. In the set of six closest compounds four compounds are toxic and two are non-toxic. It is not straightforward to justify the predictions only by looking at the set of most similar compounds. Nevertheless, the insight into the pool of compounds that represent the source of information for the model prediction may be of great help to the user who is assessing an unknown chemical.

### 3.2. Counter Propagation Models for Categorization of Endocrine Disrupters

The present study grounds on a data set of 146 compounds selected from 553 chemicals suspected to act as endocrine disrupters [33,34]. For modeling purposes the chemicals were categorized into four classes:

1. E: Endocrine disruptor—At least one study provides evidence of endocrine disruption in intact organism.
2. P: Potential Endocrine disruptor – *In vitro* data indicated potential endocrine disruption in intact organisms.
3. U: Nonendocrine disruptor – No certain evidence for non-ED. Category 3B – Some evidence are available, but the evidence is insufficient for identification.
4. N: Certain evidence for non-ED.

**Figure 1:**
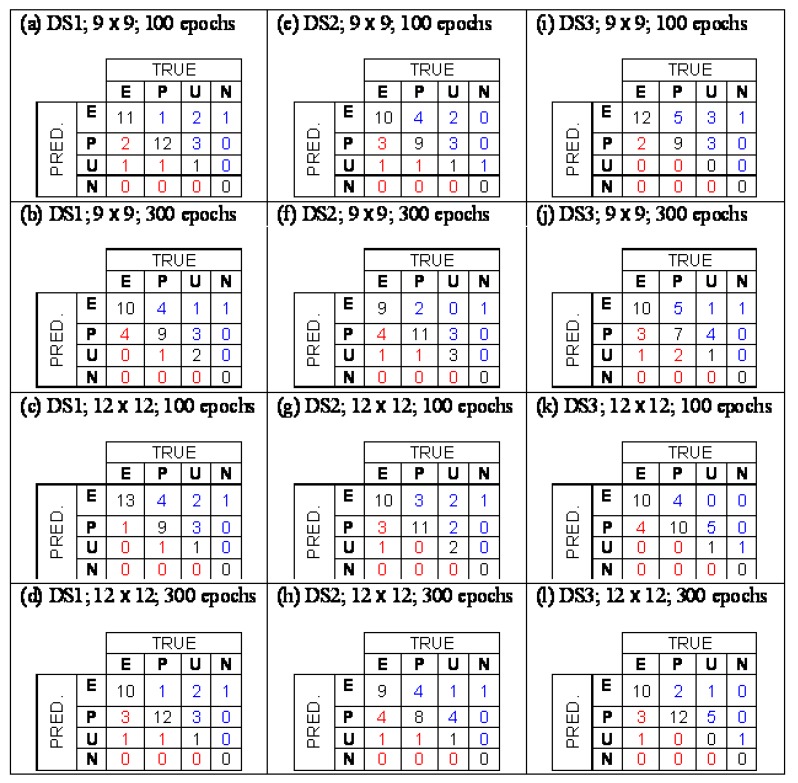
Confusion tables for different models (From [34] with permission).

The structures were described with a pool of 267 structural descriptors, including log P values. The Counter Propagation Neural Network (CP NN) was applied for modeling - method, which is often used in QSAR modeling particular for constructing of classification models [35,36]. It is a generalization of Kohonen artificial neural networks or Self Organizing Maps (SOM). Its architecture represents a network of neurons organized in 2D lattice. The training is a mapping from multidimensional descriptor space into the lattice in a way that at the end the similar objects are located close to each other. In SOM only the descriptors take part in the training while in the CP NN also propery values participate in the training. The modeling was performed for three cases, fist, all descriptors were taken into account, second, 51 descriptors were selected with the SOM method and third, 43 descriptors were selected on the same way [34]. In the classification problem the selection of the treshold for class indicating variable is essential. In the study the tresholds were optimized respecting the maximal number of correct answers. The confusion tables, which show numbers of correctly classified and misclassified structures, for selected models is shown in Figure 1. A basic problem occuring in the modeling is how to discriminate between structurally similar compounds, which belong to different classes. In our data set there are five PCB derivatives: 2,2′,3,3′,4,4′-hexachlorobiphenyl classified as U, 2,2′,3,3′,6,6′-hexachlorobiphenyl classified as P, 2,2′,4,4′,5,5′-hexachlorobiphenyl classified as E, 2,3,3′,4,4′,5-hexachlorobiphenyl classified as P, 3,3′,4,4′,5,5′-hexachlorobiphenyl classified as E. In the learning procedure the CP NN was not able to recognize the differences in the structures causing an error in the models. However, the developed classification model represents a tool for a preliminary assessment of potential endocrine disrupters. It is aimed to help the assessors to make the priority list for a large amount of chemicals that have to be tested with more expensive *in vitro* and *in vivo* methods.

### 3.3. QSAR Modeling of Relative Binding Affinity to Rat Estrogen Receptor

As the next example we present models based on the data set of 132 compounds with their relative binding affinities (RBA) to rats’ uterine estrogen receptor. The data, *i.e.*, the structures and the RBA values were collected from literature [37]. Molecular structures were described with 280 descriptors classified as constitutional, topological, geometrical, electrostatic, and quantum-chemical descriptors obtaining after optimization of structures. The octanol/water partition coefficient (log P) was added to the pool of descriptors. In reports [38,39] authors compared the results of three methods, Partial Least Square-Regression (PLS-R), CP NN and Error Back Propagation Neural Network (EBP NN). In the application of both kind of neural networks, the CP NN and the EB NN, initially the model was built using all the computed descriptors and validated with the leave-one-out procedure. Genetic algorithms have been used to successfully select the relevant descriptors. The high positive coefficient for log P and the corresponding negative contribution from the absolute number of oxygen atoms suggests that the polarity of the sample is involved in modulating the binding of the endocrine disrupter to the receptor site: specifically, less polar molecules, characterized by a high log P and by a small number of oxygen atoms, are supposed to be more favored with respect to highly polar ligands. A significant positive correlation is observed between the dependent variable and the final heat of formation of the molecule, the energy of the HOMO-1 and the maximum total interaction for a C-C bond. This issue suggests that probably the binding of the ligand to the receptor site involves a certain degree of electron transfer. High HOMO-1 energy (the second highest energy of valence electron) indicates that less bound valence electrons enhance the ligand-protein complex formation. On the other hand, the van der Waals interactions, which are present and important for the binding mechanism, are accounted for by the polarity terms described before. The best overall regression model was found to be a 50-10-1 network (521 weighted connections including bias nodes) trained for 2500 epochs with learning rate η = 0.15 and momentum μ = 0.15. This optimal BP NN model performed better that the PLS-R or CP-ANN models, with the R^2^ = 0.92 and Q^2^ = 0.71. 

### 3.4. Receptor Dependent Models

The study was performed on two data sets, both reporting the binding affinities toward natural ligand 17β-estradiol for human ER-alpha and ER-beta. The first set (‘Kuiper dataset’) consists of 60 chemicals including environmental estrogenic compounds and phytoestrogens [40]. The second set (‘Harris dataset’) contains some new compounds, in addition to those from ‘Kuiper dataset’. The reported affinities for both sets are not the same, however, a correlation between them exists [41]. The main goal of studies decribed reported in [42,43] was to compare how the inclusion of the information on receptor influences the modeling results. In the first case the molecular structures were optimized in vacuo using Merck Molecular Force Field method followed by semi-empirical AM1 optimization. In the second case the structures were optimized in a docking procedure, having considered a 3D structure of the receptor as known from the PDB database. A pool of 278 descriptors was calculated with CODESSA software. CP NN technique was used to build classification models and models for prediction of affinity. All the models were optimised for the model parameters and for the selection of descriptors with internal test set and validated by the external validation set of compounds. We analyse the selected descriptors and try to interpret them regarding the selectivity between the ER-alpha and ER-beta receptors as predicted by the two models. Common variables for alpha and beta receptor models in the first approach with the receptor-independent ligand conformations are: Relative number of N atoms, Kier Shape index-2, Minimum and Average electrophilic Reactivity index for C atom, descriptors related to H-donors and H- bonding surface area, HDCA H-donors charged surface area, HBCA H-bonding charged surface area, and Principal moment of Inertia. There is also a series of variables chosen specifically for alpha and beta models, but they appear close to each other in the Kohonen map, what means that they are similar and they aren’t receptor subtype specific. The variables specific for ER-alpha model are the following: descriptors for the polar interaction between molecules and functional group portions, such as CPSA variables, PPSA-1, Partial Positive Surface Area (sum of surface area on positive parts of molecule) and PPSA-2, and Total charge weighted CPSA. Additionally, the LUMO+1 energy, second lowest unoccupied molecular level is also specific for alpha receptor. It is interesting that among them there are the following variables: Number of S atoms and Relative number of S atom, and HOMO Energy. In the map describing the distribution of variables the variables “No. of S atoms” and “relative No. of S atoms”, are located in the area where there is no similar descriptor selected for alpha receptor; those two descriptors seem to influence the beta model only. The descriptors characterizing polar interactions may be useful to discriminate between structurally different chemical compounds that bind to the ER-alpha and ER-beta with specific interaction not only dependent on the binding pocket residues and bonds but also on the interactions around the pocket that are able to modify the size of the cavity and the affinity of the ligand for the receptor. Several studies referred the shape, dimension, and polar interaction as parameters to define the selectiveness of receptors for alpha or beta subtype. From our study we can conclude that one can not obtain a common, well selective model for ER-alpha and ER-beta binding affinities. It is better to have two separate models and apply them sequentially for the determination of ER-alpha and ER-beta binding affinities of unknown compounds. Obviously, besides few common influential descriptors, different descriptors are important for describing structure-property relationships of different receptor types. 

The comparison of the prediction results of the two approaches described in [42,43], with or without inclusion of the information on the receptor structure, demonstrates that there is no significant difference in the predictive ability of the obtained models. Although one would expect that the information about the ligand conformation in the host protein binding site would improve the model quality, obviously this improvement did not exceed the inherent error of the modeling methodology itself. The error introduced by inaccurate conformation seems to be compensated by the optimisation of model parameters and variable selection procedure. See Table 2 for details.

## 4. Conclusions

Reproductive toxicity is an important regulatory endpoint. Different regulatory agencies have defined classification schemes where the compounds are grouped according to their harm to human or wildlife. The classification is usually performed by experts on the basis of different scientific evidences. In this overview we report on some strategies for modelling potential reproductive toxicity. As first example we present the CAESAR model, which was recently placed on the Internet. Within the CAESAR project, a data mining approach has been employed using a highly verified set of compounds (all chemical structures have been double-checked, and experimental data verified in case of some unusual finding, compared to similar compounds), and adopting a wide series of chemical descriptors, reduced to the most influential ones in the modelling optimisation procedure. Different algorithms have been applied for modeling, resulting in a series of models and one with optimal performance has been implemented. The predicted value expressed in binary form (toxic/non-toxic) represents evidence, which can be used in early stage of hazard assessment of chemicals. In further examples we select the mechanism, *i.e.*, the binding to estrogen receptor. Such models are more specific and the reliable predictions within assessed confidential limits could be made. The drawback of these models is in their applicability for everyday regulatory use. Specific software and expert knowledge is required what is sometimes not available in regulatory entities. A further problem is usually caused by an imbalanced distribution of compounds in different classes. The chemicals may cause harm due to different mechanisms and at the end they are classified in the same class. This means that a general classification model indeed includes many different structure-toxicity relationships. Unfortunately, the data sets, which are used for training of models, are rather limited. Usually, more data is available for positive (toxic) compounds than for negative ones what means that the structural domain of negatives is smaller than for positives. 

## Figures and Tables

**Table 1:** Predictions of developmental toxicit for four PAH compounds using he CAESAR model. For each compound the table shows similarity indices to six most similar structures of training set. (T - toxic, NT - non-toxic).

	Anthracene	Fluorene	Fluoranthene	Triphenylene
Prediction	Tox.	NON-Tox.	Tox.	NON-Tox.
Phenyltoloxamine T	0.954	0.962		
Aminacrine NT	0.949	0.948	0.920	
Diphenylhydramine NT	0.948			
Alprazolam T	0.942		0.927	
Promethazine T	0.940	0.962		
Dotheipin T	0.936	0.957		
Imipramine T		0.954		
Amitriptyline T		0.946		
Chlorotrianisene T			0.929	0.951
Phenolphthalein T			0.925	0.947
Clomiphene T			0.915	0.952
Clotrimazole NT			0.911	0.964
Diphenadione T				0.943
Loperamide NT				0.918

**Table 2:** Performance values summary for receptor dependent and receptor independent approach.

Performances		Receptor Independent Approach				Receptor Dependent Approach			
Approach	Model	ER-α		ER-β		ER-α		ER-β	
		Var A*	Var R**	Var A*	Var R**	Var A*	Var R**	Var A*	Var R**
**Training & Test Set**	**Classification Error (%)**	7	0	7	0	5	0	12	2
	**RMS Error of Predictions of Active Compounds**	0.61	0.17	0.41	0.24	1.61	0.36	1.79	0.85
**External Set**	**Classification Error (%)**	0	0	0	0	0	18	0	0
	**RMS Error of Predictions of Active Compounds**	2.43	2.20	2.34	1.33	2.12	5.14	2.83	1.86

* All variables included in the model.

** Reduced set of selected variables included in the model.
